# *In silico* identification of promising PD-L1 inhibitors from selected indian medicinal plants for treatment of triple negative breast cancer

**DOI:** 10.1371/journal.pone.0327475

**Published:** 2025-07-10

**Authors:** Sk. Faisal Ahmed, Md. Shohel Hossain, Amalesh Mondal, Musab Shahariar, Shormila Akter Sumya, Nahid Sultan Rizu, Lamia Hasan Joarder Barsha

**Affiliations:** 1 Dawn of Bioinformatics Limited, Dhaka, Bangladesh; 2 Department of Pharmacy, Gono Bishwabidyalay, Savar, Dhaka, Bangladesh; 3 Department of Physiology, Katwa College, Katwa, Purba Bardhaman, West Bengal, India; 4 Department of Pharmacy, Islamic University, Kushtia, Bangladesh; 5 Department of Pharmacy, Primeasia University, Dhaka, Bangladesh; 6 Department of Biochemistry and Molecular Biology, Noakhali Science and Technology University, Noakhali, Bangladesh; Kwara State University, NIGERIA

## Abstract

Triple-negative breast cancer (TNBC) is the most aggressive among the breast cancer subtypes and poses unique therapeutic challenges due to its distinct characteristics like lack of specific therapeutic targets. TNBC demonstrates poor survival rate enhanced immunogenic characteristics and a more favorable tumor microenvironment than other breast cancer variants. Also, TNBC patients show elevated levels of programmed death ligand-1 (PD-L1) expression in contrast to non-TNBC patients. Binding of PD-L1 with PD-1 produces an inhibitory signal, resulting in suppression of T-cell. Therapeutic approaches utilizing immunotherapies against PD-L1 exhibit promising outcomes in the treatment of TNBC. Limitations like suboptimal efficacy, inadequate oral bioavailability, and associated immune-related adverse effects of antibody-mediated anti PD-1/PD-L1 therapies have necessitated the exploration of alternative therapeutic approaches. Thus, small molecules become an alternate option for PD-1/PD-L1 inhibition. In the present study, we have used virtual screening to identify potential phytochemicals from selected Indian medicinal plants as PD-L1 inhibitors. A total of 953 phytochemicals derived from eleven selected medicinal plants were initially screened through molecular docking using the PyRx tool. Among the 953 identified phytochemicals, the top 20 compounds exhibiting the highest binding affinities in docking study were selected for further analysis. Following comprehensive ADMET analyses, 2 compounds were ultimately identified as suitable candidates for a molecular dynamics (MD) simulation study. The study identified 4-hydroxychalcone and flavylium from *Glycyrrhiza glabra* and *Catharanthus roseus,* respectively as potential PD-L1 inhibitors with enhanced stability relative to the reference molecule. Both compounds also showed enhanced gastrointestinal absorption with no predicted cytotoxic and immunotoxic effects. Consequently, these compounds present promising candidates for novel PD-L1 inhibitor development in TNBC therapy. Further experimental investigations are necessary to facilitate their clinical translation.

## 1. Introduction

Triple-negative breast cancer (TNBC) represents the most aggressive phenotypic subtype within the spectrum of breast cancer (BC) [1]. In TNBC, the minimal cellular expression of progesterone and estrogen receptors is typically at or below 1%, while human growth factor receptor 2 expression ranges from 0 to 1 + . TNBC accounts for 15–20% of all BC cases and has the least favorable five-year overall survival (OS) rate compared to other BC subtypes [2]. TNBC is unique due to its aggressive behavior, molecular complexity in the metastatic process, and lack of effective targeted therapies [1]. Therefore, chemotherapy remains the primary choice for its treatment [3]. While chemotherapy has served as the fundamental therapeutic option for TNBC in recent decades, the treatment is linked to an elevated likelihood of distant metastasis, increased rates of metastasis, and an enhanced propensity for disease relapse, contributing to a rise in chemoresistant individuals. Consequently, extensive research endeavors have been undertaken in the last few years to explore novel therapeutic approaches for TNBC [4,5]. In contrast to other subtypes of BC, TNBC demonstrates increased immunogenicity with a notable abundance of tumor-infiltrating lymphocytes (TILs). The infiltration of TILs establishes a favorable tumor microenvironment (TME) in TNBC, the condition chooses therapeutic approaches involving immune checkpoint inhibitors (ICIs) [6,7]. Amidst the burgeoning realm of checkpoint inhibitors that block the programmed cell death protein 1/programmed death-ligand 1 (PD-1/PD-L1) interaction, these have become a promising therapeutic interest nowadays, as PD-L1 is upregulated in TNBC patients compared to those without TNBC [8]. PD-L1 attenuates the host immune response to malignant tumor cells [9]. Therefore, PD-L1 has become a promising therapeutic target for treating TNBC, particularly in metastatic conditions [1,6,10]. Most ongoing trials emphasize the integration of anti-PD-1/PD-L1 therapy with neoadjuvant chemotherapy, adjuvant therapy, or targeted therapeutic approaches [11,12]. Compared to traditional anti-tumor therapies, the use of monoclonal antibodies (mAbs) has shown superior advantages by extending progression-free survival (PFS) and eliciting strong antitumor immune responses when combined with chemotherapy, radiation, and targeted therapies [1]. In March 2019, the FDA approved the use of Atezolizumab (mAb) in combination with nab-paclitaxel as the initial ICI for BC, specifically for the treatment of adult patients with metastatic TNBC exhibiting high PD-L1 expression [11]. In 2020, pembrolizumab was approved for the treatment of metastatic TNBC patients with PD-L1 expression when used in combination with chemotherapy [13]. The phase I/II clinical trial KEYNOTE-162 assessed the safety and effectiveness of the Poly ADP-ribose polymerase (PARP) inhibitor niraparib in combination with pembrolizumab for treating patients with unresectable locally advanced TNBC or mTNBC. The results indicated that patients with PD-L1-positive tumors responded more favorably than those with PD-L1-negative tumors, with response rates of 33% and 8%, respectively [14]. In July 2021, the drug received approval for an additional use: high-risk, early-stage TNBC. This decision was based on findings from the phase-III KEYNOTE-522 trial (NCT 03036488), which showed that incorporating pembrolizumab into neoadjuvant chemotherapy enhanced pathological complete response rates and event-free survival in early-stage TNBC [15,16]. Besides that, two lncRNAs, such as UCA1 and HCP5, have not yet been recognized concerning the tumor immune response in BC but have the potential to serve as valuable biomarkers for identifying patients suitable for anti-PD-1 antibody therapy [15]. Despite their advantages over traditional methods, the use of mAbs has faced several limitations. The most significant drawback of using mAbs for immune checkpoint blockade is the moderately low response rate observed in the majority of cancer cases, typically ranging from 10% to 30% [17]. This limitation led to the FDA’s revocation of approval for Atezolizumab due to the lack of apparent clinical benefits [18]. Other limitations of ICIs includes lack of oral bioavailability, elevated manufacturing expenses, poor stability, immune-related side effects, minimal tumor penetration, and possible immunogenicity [10]. Furthermore, TNBC imposes a substantial economic burden on both healthcare systems and patients. Patients with TNBC are typically diagnosed at more advanced stages, face a worse prognosis, have a greater likelihood of recurrence, and require more hospital resources and incur higher healthcare costs than those with non-TNBC subtypes. The average annual direct medical costs per patient varied from approximately $20,000 to over $100,000 for stage I–III TNBC and from $100,000 to $300,000 for stage IV TNBC in US. Cancer recurrence resulted in a notable decline in productivity and an increased likelihood of individuals leaving the workforce. The estimated indirect costs from productivity loss varied between $207 and $1573 per patient each month [19]. On the other hand, ineffective chemotherapy and chemotherapy-induced toxicity increase the burden of treatment by requiring greater resource utilization and extended hospital stays. They often lead to undesirable side effects and long-term adverse health consequences, negatively impacting the patient’s quality of life. [1]. Those with TNBC who were young and who received chemotherapy were 10% more likely to experience financial hardship [1,20]. The average monthly costs for patients were more than $1000 in mTNBC and over $2000 in eTNBC during both the neoadjuvant and adjuvant therapy periods in the US. These financial adverse events impact treatment choice, compliance, well-being, and cancer outcomes [21,22]. Therefore, researchers have focused on exploring the potential of small-molecule inhibitors, which are characterized by an enhanced therapeutic index and oral bioavailability.

Immunotherapy based on small molecules can function in the same mechanism as mAbs do without facing the limitations [23]. Furthermore, due to their shorter pharmacokinetic half-life, small molecules have the potential to offer a superior therapeutic index, thereby improving the management of any unforeseen adverse events [24]. On the other hand, the synthetic manufacturing of small molecules is more cost-effective for patients. Small molecule inhibitors designed to target PD-L1 are categorized into two main groups: small molecules inspired by amino acids that replicate the receptor-ligand interface, and the second group consists of compounds structured on the biphenyl scaffold. The pioneering amino acid-inspired interface mimic, CA-170, emerged as the foremost orally administrable small molecule inhibitor of PD-L1 that underwent clinical trials in 2016 and is now progressing through phase-II clinical trials [24]. Researchers from Bristol-Myers Squibb (BMS) have formulated a collection of biphenyl derivatives with substitutions based on their efficacy in impeding the interaction between PD-1 and PD-L1 [24]. Notably, BMS-1001 and BMS-1166 have shown the best safety profiles and remarkable potency in inhibiting the PD-1/PD-L1 interaction [23]. But none of the small-molecule inhibitors of PD-1/PD-L1 pathway have received approval from the FDA [10,23,24]. Throughout history, plant-based bioactive compounds have been used to treat numerous diseases, including cancer. The discovery of natural phytochemicals that inhibit PD-L1 could pave the way for the development of cancer immunotherapeutics [25–27]. In this backdrop, the present study was designed to identify promising natural compounds targeting PD-L1 through virtual screening of a compound database, with the aim of advancing therapeutic strategies for TNBC.

## 2. Methodology

### 2.1. Protein preparation

The crystal structure of human PD-L1 protein (PDB ID: 5J89) was retrieved from the RCSB Protein Data Bank (https://www.rcsb.org/) with a resolution of 2.20 Å. The protein was prepared using the Discovery Studio 2024 (https://discover.3ds.com/discovery-studio-visualizer-download) by eliminating the cofactors, ligands, water molecules, and metal ions bound to the protein. Energy minimization was performed using SWISS PDB Viewer (https://spdbv.vital-it.ch/) to stabilize the protein structure [28].

### 2.2. Ligand retrieval and preparation

In this study, 11 Indian medicinal plants (Table 1) were chosen for anti-PD-L1 drug development based on a literature study [29–35]. The PubChem Compound Identities (CIDs) of all the bioactive metabolites of these 11 plants were retrieved from the IMPPAT database (https://cb.imsc.res.in/imppat/). Following the removal of duplicates, 953 compounds (S1 Table) from these 11 plants were selected for subsequent investigation. The IMPPAT database (https://cb.imsc.res.in/imppat/) is a repository of phytochemical data on Indian medicinal plants [36]. The database utilizes cheminformatics methodologies to evaluate its physicochemical and drug-like properties, employing various scoring methodologies. The 3D structures of the ligands were retrieved from the PubChem database (https://pubchem.ncbi.nlm.nih.gov/) in SDF format [37,38]. We have used Open Babel (https://sourceforge.net/projects/openbabel/) to generate a ligand library [38]. Energy minimization of the ligand compounds was performed using the Universal Force Field (UFF) and the Conjugate Gradients algorithm of PyRx [39].

**Table 1:** Traditional uses and therapeutic potentials of the selected plants.

Plant	Traditional uses against different ailments	Promising pharmacological activities	Reference(s)
*Moringa oleifera*	Skin infections, sore throat, swelling	Anti-inflammatory, antioxidant, anti-tumor, anticancer, immunomodulatory	[40]
*Glycyrrhiza glabra*	Oxidative stress, upper respiratory infections, sore throat, conjunctivitis	Antioxidant, anti-inflammatory, immunoregulatory, antineoplastic	[29]
*Alternanthera sessilis*	Oxidative stress	Anti-inflammatory, antioxidant, anticancer	[41]
*Rauwolfia serpentina*	Snake bites, fever, and bug stings	Anti-breast cancer, anti-prostate cancer, anti-tumor	[42]
*Cassia fistula*	Pain, edema, swelling, inflammation	Antioxidant, anti-inflammatory, anti-tumor	[34]
*Withania somnifera*	Painful swelling, edema, arthritis	Immunomodulatory, antioxidant, anti-inflammatory, anti-tumor	[32]
*Annona muricate*	Fever, inflammation, arthritis	Anticancer, antioxidant, anti-inflammatory	[31]
*Hibiscus rosa-sinensis*	Fever, inflammation, asthma	Anti-inflammatory, antioxidant, anticancer, immunomodulatory	[35]
*Phyllanthus emblica*	Jaundice, inflammation	Anti-breast cancer, antioxidant, anti-inflammatory, immunity enhancer	[30]
*Allium sativum*	Fever, tumor	Anticancer, anti-inflammatory, antioxidant	[43]
*Catharanthus roseus*	Throat, stomach, oesophageal cancer	Anticancer, antioxidant	[33,44]

### 2.3. Virtual screening and molecular docking

The molecular docking method finds more potent, selective, and efficient drug candidates [44]. Accordingly, virtual screening and blind molecular docking techniques were performed to select a limited number of ligand compounds from the ligand library with desired biological functions, capable of interacting with the binding pocket of the target protein (receptor) [39,45] The ligand compounds were docked against the target protein PD-L1 using PyRx, an open-access virtual screening tool [46]. A molecular docking program, AutoDockVina under the PyRx, was employed for this docking purpose and to estimate the binding affinities of docked complexes. With the help of the calculation of the value of energy minimization and binding energy, molecular docking predicts possible drug-target interactions [47]. During the docking process, all PyRx configurations and parameters were kept at default. The 3D coordinates of the grid box were set as per the co-crystal ligand (active binding site of the reference drug) for structure-based virtual screening. The docked complexes were visualized and analyzed using the BIOVIA visualizer of Discovery Studio 2024 [44].

### 2.4. ADMET analysis

The top twenty ligand compounds were subjected to pharmacokinetic and drug-likeness evaluation using the SwissADME (www.swissadme.ch) web server, a widely used tool for in silico prediction of ADME (Absorption, Distribution, Metabolism, and Excretion) parameters [47]. Pharmacokinetic properties (PKs) like physicochemical properties, lipophilicity, water solubility Log S (ESOL), pharmacokinetics, drug-likeness rules (Lipinski), and medicinal chemistry (PAINS, Synthetic accessibility) of drugs hold immense significance in pharmacological research for understanding how drugs behave within the body [48]. The Canonical SMILES of each compound were utilized as input to retrieve data on physicochemical properties (e.g., molecular weight, hydrogen bond donors and acceptors), lipophilicity (iLOGP), water solubility (Log S), pharmacokinetic parameters (e.g., gastrointestinal absorption), drug-likeness criteria (e.g., Lipinski’s Rule of Five, bioavailability score), and medicinal chemistry filters (e.g., synthetic accessibility). These properties are critical for assessing the oral bioavailability and therapeutic potential of small molecules [37,47,48].

To ensure the safety profile of the selected compounds, toxicity predictions were conducted using admetSAR 2.0 (http://lmmd.ecust.edu.cn/admetsar2) and Protox-III (https://tox-new.charite.de/) web servers [49,50] web servers. Both servers utilize canonical SMILES as input to predict key toxicological endpoints. The admetSAR 2.0 provides insights into parameters such as AMES mutagenicity, acute oral toxicity, LD50 of Rat, and hERG channel inhibition, while Protox-III evaluates potential carcinogenicity, cytotoxicity, and immunotoxicity of selected ligand compounds.

### 2.5. Molecular dynamics simulation

Molecular dynamics (MD) simulation is one of the most efficient and frequently used computer techniques for studying the dynamics of macromolecules in biological systems [51]. Therefore, MD simulation was performed in our study to assess the stability of ligand-receptor complexes obtained from molecular docking. We have used YASARA Structure (Product name: YASARA Dynamics) (http://www.yasara.org/products) for MD simulation. MD run was conducted for the ligand and control molecules for 100 ns with 401 snapshots [52]. The homology modeling was carried out using YASARA in many stages. YASARA first determines the modeling parameters and homology modeling target that the macro specifies [53]. The scene mode was then subjected to MD simulations using the default settings of the YASARA Structure macro for MD run (http://www.yasara.org/md_run.mcr) [54]. The MD simulation was conducted using the AMBER (Assisted Model Building with Energy Refinement) force field under the following conditions: a temperature of 298 K, a pressure of 1 bar, Coulomb electrostatics with a cut-off of 7.86, 0.9% NaCl (to neutralize charges), solvent density of 0.997, pH 7.0, 1-fs time phases, periodic boundaries, and all mobile atoms [52,53]. MD simulation was performed using the same methodology as in previous studies [28]. The interaction energy, the root mean square deviation (RMSD), the root mean square fluctuation (RMSF), the radius of gyration (RG), solvent accessible surface area (SASA) and the total number of hydrogen bonds were evaluated from trajectory files.

### 2.6. MM-PBSA analysis/energy of binding analysis method

Considering the strong correlation of structural changes of the receptor upon ligand binding, the Molecular Mechanics Poisson-Boltzmann Surface Area (MM-PBSA) method was used to calculate the binding affinity of protein-ligand interaction, where a more positive energy value indicates a more favorable binding interaction [55]. Binding free energy assessment methodologies serve as a valuable tool for rescoring docked ligand-protein complexes, providing more accurate estimates of ligand-protein binding affinities compared to basic scoring functions. Different ligand orientations generated by docking software were considered as the initial ligand-receptor coordinates for MD simulations to achieve this goal. The MD simulation was further used for MM-PBSA assessment. The most favorable ligand orientation was subsequently determined by comparing the best binding free energy that was obtained [56,57]. The stable area of the three PD-L1 complexes was used to generate a 100 ns MD trajectory for MM-PBSA calculations. The binding energy components were measured using the MM-PBSA technique in the YASARA simulator (YASARA Biosciences). The g_mmpbsa tool calculates the binding energy of the protein-ligand complex using the following equation.


ΔGBinding = GComplex − (GProtein + GLigand)

Where ∆GBinding denotes total binding energy, GComplex denotes the total free energy of binding complex, GProtein and GLigand symbolize the total free energy of the three molecules that are attached to PD-L1, correspondingly [52].

### 2.7. Principle component analysis

A multivariate statistical method, principal component analysis (PCA), was used to analyze collective motion of the protein-bound ligands during the MD simulation over a 100 ns covariance matrix of backbones atoms was created to conduct the PCA. Various structural and energy information, such as bond lengths, bond angles, dihedral angles, planarity, Van der Waals energies, and electrostatic energies, can be utilized to elucidate the distinctions among various categories [58].Minitab 18 statistical software was used for analysis and plot generation. This tool mitigates complexities linked to elucidating extensive data sets by decreasing their dimensionality and minimizing data loss [59]. The global motion of receptor-ligand complexes was observed by analyzing two principal components, PC1 (projection on eigenvector 1) and PC2 (projection of eigenvector 2).

## 3. Results

### 3.1. 3D grid box preparation

PD-L1 is upregulated in TNBC, especially in metastatic conditions. Therefore, it is necessary to inhibit the expression of PD-L1 through the development of suitable drug candidates. For this purpose, we have defined a grid box around the active residues within the binding pocket of PD-L1. The grid box dimensions (Å) were set as center (x = 16.6413, _y = −11.5197, _z = 181.8531), with size x = 39.8515712214, y = 56.774720726, and z = 52.5295007324.

### 3.2. Virtual screening and molecular docking analyses

We have screened 953 potential bioactive phytochemicals from the ligand library using PyRx. The top 20 compounds were selected for further analysis based on binding affinities between receptor and ligands in terms of binding energy (docking score) in kcal/mol (S1 Table). We have also docked BMS-1166 (PubChem CID-118434635), a control inhibitor of PD-L1, against the target receptor to compare the binding affinities of the selected compounds and observed to have docking score of −7.9 kcal/mol. The selected compounds exhibited higher binding affinities than the control compound, with docking scores ranging from –10.4 to –8.8 kcal/mol, indicating their potential as PD-L1 inhibitors (Table 2). The docked models in PDB format of these 20 protein-ligand complexes are given S1 File.

**Table 2:** List of top 20 phytochemicals, along with the control compound, including their corresponding PubChem CID, chemical name, and docking score (kcal/mol).

Serial No	Compounds (PubChem ID)	Compound Name	Docking Score (kcal/mol)
1	CID-10881804	Kanzonol B	−10.4
2	CID-452707	Gallotannin	−10.2
3	CID-5317764	Glycyrrhisoflavone	−9.9
4	CID-5282361	4-Hydroxychalcone	−9.9
5	CID-72304	Isoflavone	−9.8
6	CID-16398538	Prunetin 4’-O-glucoside	−9.6
7	CID-145858	Flavylium	−9.5
8	CID-637247	alpha,alpha’-Dihydro-3,5,4’-trihydroxy-4,5’-diisopentenylstilbene	−9.5
9	CID-5889042	Isobavachromene	−9.3
10	CID-14106343	Somniferine	−9.3
11	CID-161345	Isoreserpiline	−9.2
12	CID-11597485	Glychionide A	−9.1
13	CID-11349817	licoflavone B	−9.1
14	CID-5320092	Neoisoliquiritine	−9.1
15	CID-72281	Hesperetin	−9
16	CID-785868	Methylenedioxybenzoyl ethyl PABA	−9
17	CID-439246	Naringenin	−9
18	CID-480865	Licoricidin	−8.9
19	CID-5280537	N-Trans-feruloyltramine	−8.9
20	CID-10026486	Asperglaucide	−8.8
21	CID-118434635 (Control)	BMS-1166	−7.9

### 3.3. Interaction analyses of receptor-ligand and receptor-control complexes

The interactions analysis of the selected docked complexes was carried out using Discover Studio 2024. The various types of interactions mainly observed in these complexes were conventional hydrogen bond, carbon-hydrogen bond, Pi-Sigma, Pi-Pi Stacked, Pi-Pi T-shaped, Pi-Alkyl, Pi-Sulfur, Amide-Pi Stacked, and Pi-Cation (Table 3). These interactions are responsible for the extent of binding affinities and docking scores of docked complexes [60].

**Table 3:** Non-bond interactions of PD-L1 with the top 20 compounds and the control compound BMS-1166 for comparative analysis.

Sl. No.	Compounds (PubChem ID)	Interactions
1.	CID-10881804	**Conventional Hydrogen Bond:** A: PHE19, A: ASP122, B: GLN66
		**Pi-Sigma:** A: ALA121
		**Pi-Pi Stacked:** B: TYR56
		**Alkyl:** A: MET115, B: MET115, A: ALA121, B: ALA121
		**Pi-Alkyl:** B: VAL68, B: MET115, B: TYR123
2.	CID-452707	**Conventional Hydrogen Bond:** A: ALA121, A: TYR123, A: LYS124, A: ARG125, B: TYR56, B: ILE54, B: GLU58, B: GLN66, B: GLU71, B: MET115
		**Carbon Hydrogen Bond:** A: ASP122
		**Pi-Sigma:** A: ALA121
		**Pi-Alkyl:** B: VAL68, B: MET115
3.	CID-5317764	**Conventional Hydrogen Bond:** A: ASP122, B: ILE116
		**Pi-Anion:** A: ASP122
		**Pi-Sigma:** A: ALA121, A: ASP122, B: MET115
		**Pi-Sulfur:** A: MET115
		**Pi-Pi Stacked:** B: TYR56
		**Alkyl:** B: VAL68
		**Pi-Alkyl:** A: ALA121, B: ALA121, B: MET115
4.	CID-5282361	**Conventional Hydrogen Bond:** B: ILE116
		**Pi-Sigma:** B: ALA121
		**Pi-Pi Stacked:** B: TYR56
		**Pi-Pi T-shaped:** A: TYR56
		**Pi-Alkyl:** A: MET115, A: ALA121
5.	CID-72304	**Pi-Sigma:** A: ALA121
		**Pi-Sulfur:** A: MET115, B: MET115
		**Pi-Pi Stacked:** B: TYR56
		**Pi-Pi T-shaped:** A: TYR56
		**Amide-Pi Stacked:** A: ALA121
		**Pi-Alkyl:** A: MET115, B: MET115, A: ALA121, B: ALA121
6.	CID-16398538	**Conventional Hydrogen Bond:** B: GLN66
		**Pi-Anion:** A: ASP122
		**Pi-Sigma:** A: ASP122, B: MET115
		**Pi-Sulfur:** A: MET115, B: MET115
		**Pi-Pi Stacked:** B: TYR56
		**Amide-Pi Stacked:** A: ASP122
		**Alkyl:** B: ALA121, A: MET115
		**Pi-Alkyl:** A: TYR56, A: ALA121, B: ALA121
7.	CID-145858	**Pi-Sulfur:** A: ALA121
		**Pi-Pi Stacked:** A: MET115
		**Pi-Pi T-shaped:** B: TYR56
		**Amide-Pi Stacked:** B: ALA121, B: ASP122
		**Pi-Alkyl:** A: ALA121, B: MET115, B: ALA121
8.	CID-637247	**Pi-Sigma:** A: TYR56, A: ALA121
		**Alkyl:** A: ILE54, A: MET115, B: ALA121
		**Pi-Alkyl:** B: VAL68, B: MET115
9.	CID-5889042	**Pi-Anion:** A: ASP122
		**Pi-Sigma:** A: ALA121
		**Alkyl:** B: VAL68
		**Pi-Alkyl:** B: MET115
10.	CID-14106343	**Conventional Hydrogen Bond:** A: ARG125
		**Carbon Hydrogen Bond:** A: ARG125
		**Pi-Cation:** A: ARG125
		**Alkyl:** A: ARG125
11.	CID-161345	**Conventional Hydrogen Bond:** A: ARG125, B: SER79
		**Carbon Hydrogen Bond:** A: TYR123, B: HIS78
		**Pi-Sigma:** B: HIS78
		**Alkyl:** A: LYS124, A: ARG125, B: VAL76 Pi-Alkyl: A: TYR123
12.	CID-11597485	**Conventional Hydrogen Bond:** B: GLU58, B: ASP61, B: ASN63
		**Pi-Anion:** A: ASP122
		**Pi-Donor Hydrogen Bond:** B: GLN66
		**Pi-Pi Stacked:** B: TYR56
		**Pi-Alkyl:** A: ALA121, B: MET115
13.	CID-11349817	**Conventional Hydrogen Bond:** B: PHE19, B: ALA121
		**Pi-Cation:** B: LYS124
		**Pi-Sigma:** A: VAL76, B: LYS124
		**Alkyl:** A: VAL76, A: VAL68, B: ALA18
		**Pi-Alkyl:** A: TYR56, A: VAL76, B: ALA18
14.	CID-5320092	**Conventional Hydrogen Bond:** A: TYR123, B: GLU58
		**Pi-Anion:** A: ASP122
		**Pi-Sigma:** A: ALA121
		**Pi-Pi Stacked:** B: TYR56
		**Pi-Alkyl:** B: ILE54, B: MET115
15.	CID-72281	**Conventional Hydrogen Bond:** A: ALA121
		**Pi-Sigma:** A: ASP122
		**Pi-Sulfur:** B: MET115
		**Pi-Pi Stacked:** B: TYR56
		**Alkyl:** A: ALA121, B: ILE54, B: MET115
		**Pi-Alkyl:** B: TYR56, A: ALA121
16.	CID-785868	**Pi-Sulfur:** A: MET115, B: MET115
		**Pi-Pi Stacked:** B: TYR56
		**Alkyl:** A: MET115, B: ALA121
		**Pi-Alkyl:** A: TYR56, A: ALA121
17.	CID-439246	**Conventional Hydrogen Bond:** A: ASP122, B: GLN66
		**Pi-Anion:** A: ASP122
		**Pi-Sigma:** A: ALA121, A: ASP122
		**Pi-Sulfur:** B: MET115
		**Pi-Pi Stacked:** B: TYR56
		**Pi-Alkyl:** A: MET115, B: MET115, B: ALA121
18.	CID-480865	**Pi-Anion:** A: ASP122
		**Pi-Sigma:** A: TYR56, A: ALA121
		**Pi-Sulfur:** B: MET115
		**Pi-Pi Stacked:** B: TYR56
		**Alkyl:** A: MET115, A: ILE54, A: ALA121, B: ALA121, B: ILE54, B: VAL68
		**Pi-Alkyl:** B: TYR56, B: TYR123
19.	CID-5280537	**Conventional Hydrogen Bond:** A: TYR123, A: LYS124, A: ARG125, B: ASP61
		**Pi-Anion:** B: GLU58
		**Pi-Pi Stacked:** A: TYR123
		**Alkyl:** A: LYS124, A: ARG125
		**Pi-Alkyl:** A: TYR123, B: VAL68
20.	CID-10026486	**Conventional Hydrogen Bond:** A: ASP122, B: TYR56, B: ASN63, B: GLN66
		**Pi-Cation:** A: LYS124, A: ARG125
		**Pi-Pi Stacked:** B: TYR56
		**Pi-Pi T-shaped:** A: TYR123
		**Pi-Alkyl:** A: ALA121, A: LYS124, B: MET115, B: VAL68
21.	CID-118434635 (Control)	**Conventional Hydrogen Bond:** B: ARG125
		**Carbon-Hydrogen Bond:** B: ASP122
		**Pi-Cation:** B: LYS124
		**Pi-Sigma:** A: VAL76
		**Pi-Pi T-shaped:** B: TYR123
		**Pi-Alkyl:** A: HIS78, A: VAL76

### 3.4. ADMET analysis

The ADME properties of the top 20 ligand compounds, along with the control compound BMS-1166, were predicted using the SwissADME web server (https://www.swissadme.ch). Drug-likeness was assessed based on Lipinski’s Rule of Five, which includes criteria such as molecular weight < 500 Da, hydrogen bond acceptors < 10, hydrogen bond donors < 5, and iLogP < 5 [45]. As presented in Table 4, five compounds (CID-452707, CID-637247, CID-14106343, CID-11597485 and CID-5320092) that violated lipnski rule of 5, with compound Gallotannin (CID 452707) exhibiting the highest number of violations (three). Nonetheless, the majority of compounds, including those with violations, met the oral bioavailability threshold (log P_o/w_ < 5), indicating their potential for drug-like behavior.

**Table 4:** Prediction of pharmacokinetic properties of top 20 compounds along with the control compound.

Sl. No.	Compounds (PubChem ID)	Physicochemical Properties						Lipophilicity	Water solubility	Pharmacokinetics	Drug-likeness		Medicinal Chemistry
		MW	Heavy Atoms	Aroma. heavy atoms	Rotatable bonds	H-bond acceptors	H-bond donors	LOG P_o/w_ (iLOGP)	Log S (ESOL)	GI Absorption	Lipinski (Violations)	Bioavailability Score	Synthetic accessibility
1	CID-10881804	322.35	24	12	3	4	2	3.1	−4.81	High	0	0.55	3.48
2	CID-452707	636.47	45	18	10	18	11	0.78	−3.65	Low	3	0.17	5.32
3	CID-5317764	354.35	26	16	3	6	4	2.56	−4.97	High	0	0.55	3.53
4	CID-5282361	224.25	17	12	3	2	1	2.24	−3.27	High	0	0.55	2.29
5	CID-72304	222.24	17	16	1	2	0	2.51	−3.85	High	0	0.55	2.82
6	CID-16398538	446.4	32	16	5	10	5	2.86	−3.4	Low	0	0.55	5.15
7	CID-145858	207.25	16	16	1	1	0	−0.76	−4.01	High	0	0.55	2.78
8	CID-637247	366.49	27	12	7	3	3	4.04	−6.35	High	1	0.55	3.11
9	CID-5889042	322.35	24	12	3	4	2	3.06	−4.81	High	0	0.55	3.54
10	CID-14106343	608.68	45	12	3	9	2	3.71	−5.26	High	1	0.55	7.32
11	CID-161345	412.48	30	9	4	6	1	3.65	−4.05	High	0	0.55	4.87
12	CID-11597485	446.36	32	16	4	11	6	0.86	−3.41	Low	2	0.11	5.12
13	CID-11349817	390.47	29	16	5	4	2	4.04	−6.32	High	0	0.55	3.93
14	CID-5320092	418.39	30	12	6	9	6	2.18	−2.72	Low	1	0.55	4.92
15	CID-72281	302.28	22	12	2	6	3	2.24	−3.62	High	0	0.55	3.22
16	CID-785868	313.3	23	12	6	5	1	2.99	−4.09	High	0	0.55	2.42
17	CID-439246	272.25	20	12	1	5	3	1.75	−3.49	High	0	0.55	3.01
18	CID-480865	424.53	31	12	6	5	3	3.98	−6.41	High	0	0.55	4.39
19	CID-5280537	313.35	23	12	7	4	3	2.58	−3.03	High	0	0.55	2.55
20	CID-10026486	444.52	33	18	13	4	2	3.49	−4.95	High	0	0.55	3.65
21	CID-118434635 (Control)	641.11	46	24	10	9	2	4.81	−5.81	Low	1	0.55	5.22

The top 20 ligand compounds were then subjected to toxicological profiling using the admetSAR 2.0 web server. As summarized in Table 5, all selected ligands, similar to the control compound, were predicted to be weak inhibitors of the hERG channel and tested negative for AMES mutagenicity. Most compounds were classified as toxicity class III, indicating low toxicity [61]. Further evaluation of endpoints such as carcinogenicity, immunotoxicity, and cytotoxicity was conducted using the ProTox-III web server [47]. While none of the compounds, including the control, were predicted to be carcinogenic, several exhibited immunotoxic and cytotoxic potential. Based on comprehensive ADME and toxicity assessments, two lead compounds- CID 5282361 and CID 145858 were selected for further analysis.

**Table 5:** Predicted toxicity profiles of the top 20 compounds and the control compound using admetSAR 2.0 and ProTox-III web servers.

Serial NO	Compound (PubChem ID)	Acute Oral Toxicity	LD50 of Rat (mol/kg)	Human Ether-a-go-go-Related Gene Inhibition	AMES Toxicity	Carcinogenicity (Three-class)	Immunotoxicity	Cytotoxicity
1	CID-10881804	III	2.5931	Weak	No	No	Active	Inactive
2	CID-452707	III	2.6316	Weak	No	No	Inactive	Inactive
3	CID-5317764	III	3.1415	Weak	No	No	Active	Inactive
4	CID-5282361	III	1.5456	Weak	No	No	Inactive	Inactive
5	CID-72304	II	3.3637	Weak	No	No	Inactive	Active
6	CID-16398538	III	2.2498	Weak	No	No	Active	Inactive
7	CID-145858	III	2.5726	Weak	No	No	Inactive	Inactive
8	CID-637247	III	2.3309	Weak	No	No	Inactive	Inactive
9	CID-5889042	III	2.7438	Weak	No	No	Active	Inactive
10	CID-14106343	III	3.2357	Weak	No	No	Active	Inactive
11	CID-161345	III	2.9572	Weak	No	No	Active	Inactive
12	CID-11597485	II	2.7357	Weak	No	No	Inactive	Inactive
13	CID-11349817	III	3.0230	Weak	No	No	Active	Inactive
14	CID-5320092	III	2.3517	Weak	No	No	Active	Inactive
15	CID-72281	III	3.1455	Weak	No	No	Active	Inactive
16	CID-785868	III	2.2636	Weak	No	No	Inactive	Inactive
17	CID-439246	II	3.5110	Weak	No	No	Inactive	Active
18	CID-480865	III	3.1466	Weak	No	No	Active	Inactive
19	CID-5280537	III	2.0305	Weak	No	No	Active	Inactive
20	CID-10026486	III	1.9718	Weak	No	No	Inactive	Inactive
21	CID-118434635 (Control)	III	2.7975	Weak	No	No	Inactive	Active

**Fig 1:**
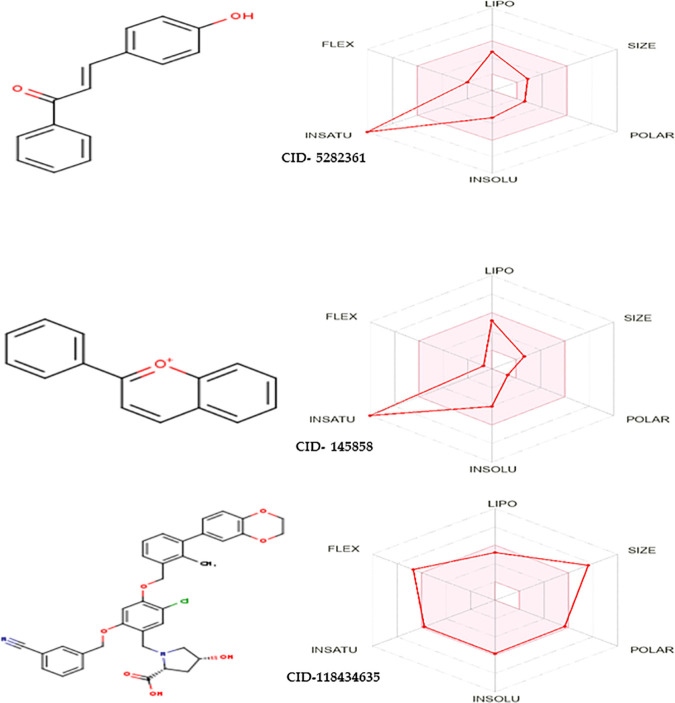
Swiss-ADME Radar Chart of selected lead compounds (CID- 5282361 and CID- 145858) and the control compound (CID- 118434635).

Furthermore, Swiss-ADME was employed to visualize the drug-likeness of final two lead compounds using radar charts. Swiss-ADME represents drug-likeness of lead compounds as a radar chart that determines whether a certain molecule is a viable candidate for a drug. In the radar chart, each peak defines different drug properties, and the pink zone denotes the optimal range for each property of the drug. In this study, almost all the drug properties of final two selected lead compounds floated within the pink zone, indicating their potency as drugs (Fig 1) [48]. The drug-likeness of the control compound was also represented as a radar chart in Fig 1.

### 3.5 Molecular docking pose assessment

**Fig 2:**
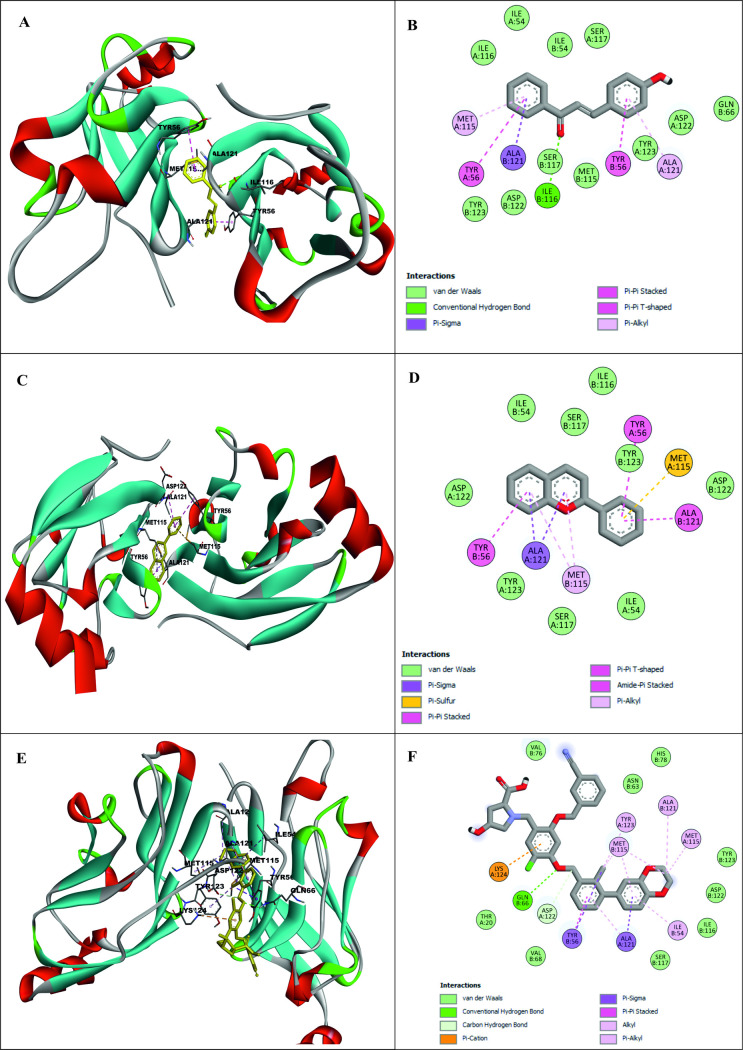
Structural representations of PD-L1 complexes with two lead compounds (CID-5282361 and CID-145858) and a control compound. Panels **(A)**, **(C)**, and (E) depict the selected binding poses of PD-L1 in complex with CID-5282361, CID-145858, and the control compound, respectively. Panels **(B)**, **(D)**, and (F) illustrate the corresponding 2D interaction diagrams of PD-L1 with CID-5282361, CID-145858, and the control compound, respectively.

Subsequent to the molecular interaction analysis, the potential binding pose of the two selected compounds were assessed and similar kind of binding pose was demonstrated by these two compounds comparing with the binding pose of control compound, suggesting a conserved binding orientation (Fig 2A, 2C and 2E). The molecular interactions of PD-L1 with these two lead compounds and with control compound were shown in Fig 2B, 2D and 2F, respectively. Subsequently, molecular dynamics (MD) simulations were performed to further validate the stability of these predicted binding conformations (48).

### 3.6. Dynamic behavior analyses of ligand-receptor complexes by molecular dynamics simulation

For investigation of the inhibitory mechanism of PD-L1, its alteration of dynamic behavior upon ligand binding was assessed through MD simulation [62]. For this purpose, a 100 ns simulation was run for both the receptor-ligand complexes and compared the outcome to that of the receptor-control compound (BMS-1166) complex.

#### 3.6.1. Root means square deviation (RMSD) analyses.

**Fig 3:**
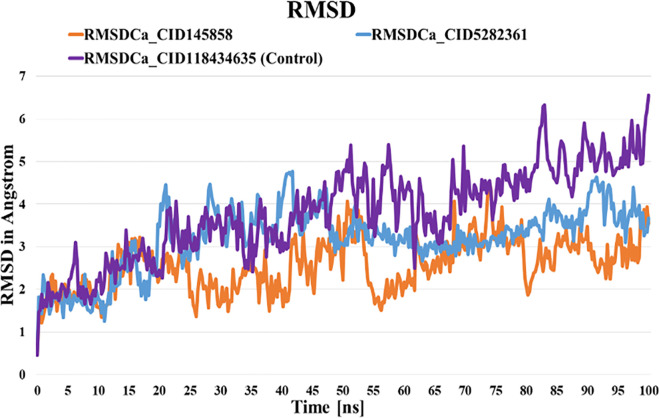
Root Means Square Deviation (RMSD) of ligand-protein complexes during 100 ns simulation.

**Fig 4:**
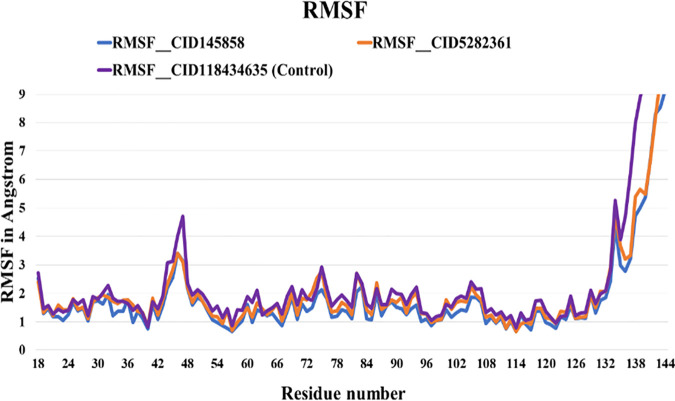
The Root Mean Square Fluctuation (RMSF) of protein residues, calculated from the average RMSF of the atoms constituting the residues.

We used the RMSD to check the stability of the ligand protein complexes [63]. The average solute RMSD of PD-L1 was calculated (Fig. 4). The RMSD of average ligand movement was determined after superposing the two ligands (CID145858 and CID-5282361) and control compound (BMS-1166) separately on the PD-L1 receptor and found the values of 1.663 Å, 1.575 Å, and 4.776 Å, respectively. The 100 ns simulation showed that the compounds were stable, not exceeding the 4 Å limit. In contrast, the ligands, CID-145858 and CID-5282361, exhibited optimal range behavior, and their values were likewise lower than those of the control compound, indicating that the two ligand-receptor complexes were more stable (Fig 3).

#### 3.6.2. Root Means Square Fluctuation (RMSF) analyses.

The thermodynamic motions of the protein residues were measured by calculating the Root Means Square Fluctuation (RMSF). This ascertains the amount of residual vibration in PD-L1 upon ligand binding. High RMSF values reveal greater flexibility, whereas low RMSF values show limitations on residue displacement throughout the 100 ns MD simulation, leading to a decrease in flexibility [64]. By analyzing the impact of ligand binding on protein flexibility, the highest fluctuations were observed with time within residues 42–54 and 132–144. On the other hand, complexes of two ligands (CID-145858 and 5282361) and control (BMS-1166) with the receptor showed average fluctuations of 1.8056 Å, 2.006 Å, and 2.169 Å, respectively.. Conversely, the CID-145858 and CID-5282361 molecules exhibited ideal range behavior, and their values were also lower than those of the control compound, suggesting a greater binding probability (Fig 4).

#### 3.6.3. Radius of Gyration (RG) analyses.

**Fig 5:**
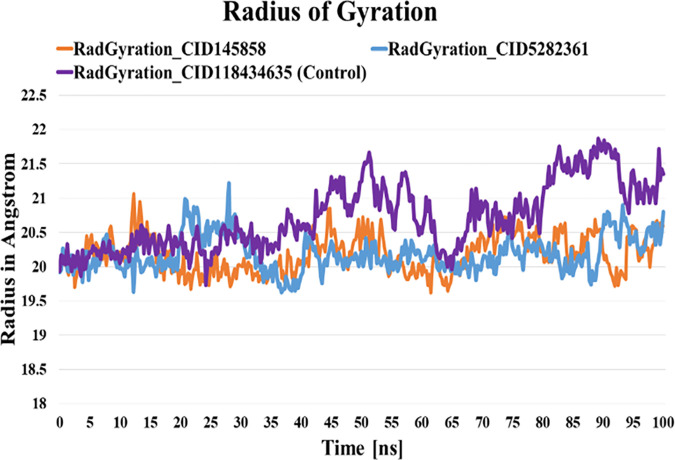
Analyses of radius of gyration of the backbone structure of PD-L1 complexes with BMS-1166, CID-145858, and 5282361 compounds over the 100 ns simulation.

RG determines a protein’s compactness and folding behavior based on its tertiary structure and overall conformational state [62]. During simulation, when achieving a stable protein structure, the fluctuation rate becomes a laser [51,62,63]. We have assessed the stability of PD-L1-CID-145858, PD-L1-CID-5282361, and PD-L1-BMS-1166 complexes by calculating the RG for each. The average RG values for the PD-L1-CID-145858 and PD-L1-CID-5282361 complexes were 20.18 Å and 20.184Å, respectively, with higher fluctuations of 21.064 Å and 21.221 Å. The control complex, PD-L1-BMS-1166, exhibited an average RG of 20.72 Å, with a greater fluctuation of 21.868Å (Fig 5). Minimal structural deviations were observed in the PD-L1-CID-145858 and PD-L1-CID-5282361 complexes compared to the control, suggesting overall stability throughout the simulation trajectory.

#### 3.6.4. Solvent-accessible surface area (SASA) analyses.

**Fig 6:**
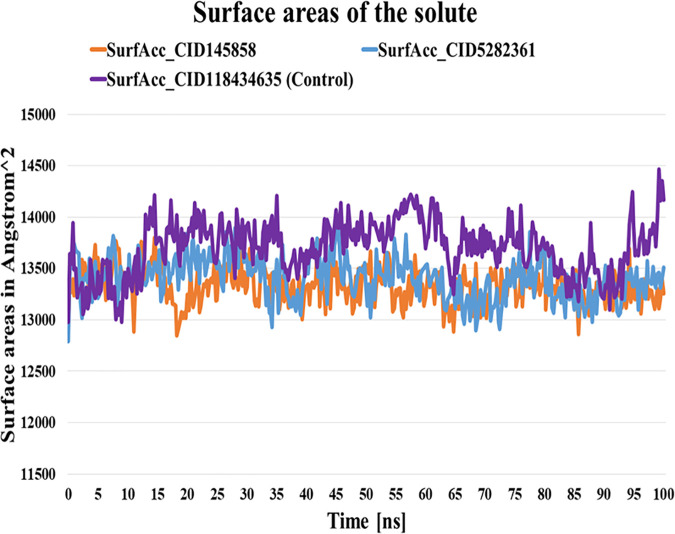
SASA analysis of the structure of PD-L1 complexes with BMS-1166, CID-145858, and 5282361 compounds over the 100 ns simulation.

SASA is a computational approach used to analyze the expansion of the ligand-protein complex’s surface area, which may undergo modifications due to biological interactions between the adsorbate and the solid surface. Alterations in the atomic structure of material surfaces can lead to surface reconstruction, significantly influencing protein conformation [65]. The control compound (BMS-1166) exhibits an average SASA value of 13703.144 Å, whereas the average values for the two selected compounds are 13326.344 Å (CID-145858) and 13407.143Å (CID-5282361), respectively. This indicates that the top two compounds have lower SASA values compared to the control. As no significant fluctuations were observed throughout the simulation period, protein expansion remains within the optimal range following binding of the top two compounds (Fig 6).

#### 3.6.5. Hydrogen bond analyses.

**Fig 7:**
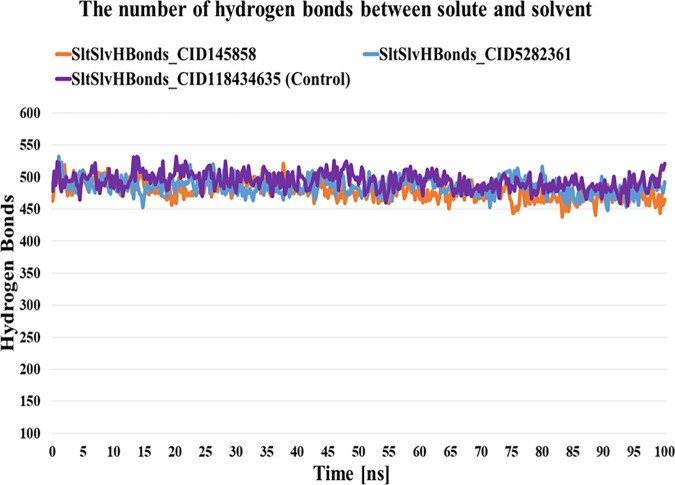
Analyses of Hydrogen bonds between solvent and solute over a 100 ns simulation.

Analyses of hydrogen (H) bonds are important because they bind the ligand to the target protein and control drug selectivity, metabolic processes, and adsorption [66]. In the present study, we calculated the total number of hydrogen bonds of three complexes (CID-145858, 5282361, and BMS-1166) in the solute and between the solute and the solvent over a 100-ns simulation. We observed 164–208, 163–209, and 163–205 numbers of H-bonds of the respective complexes in the solute. Additionally, the number of H-bonds between the solute and the solvent was 439–521, 448–532, and 456–532 for the respective complexes (Fig 7).

### 3.7. Principal component analysis (PCA) analyses

**Fig 8:**
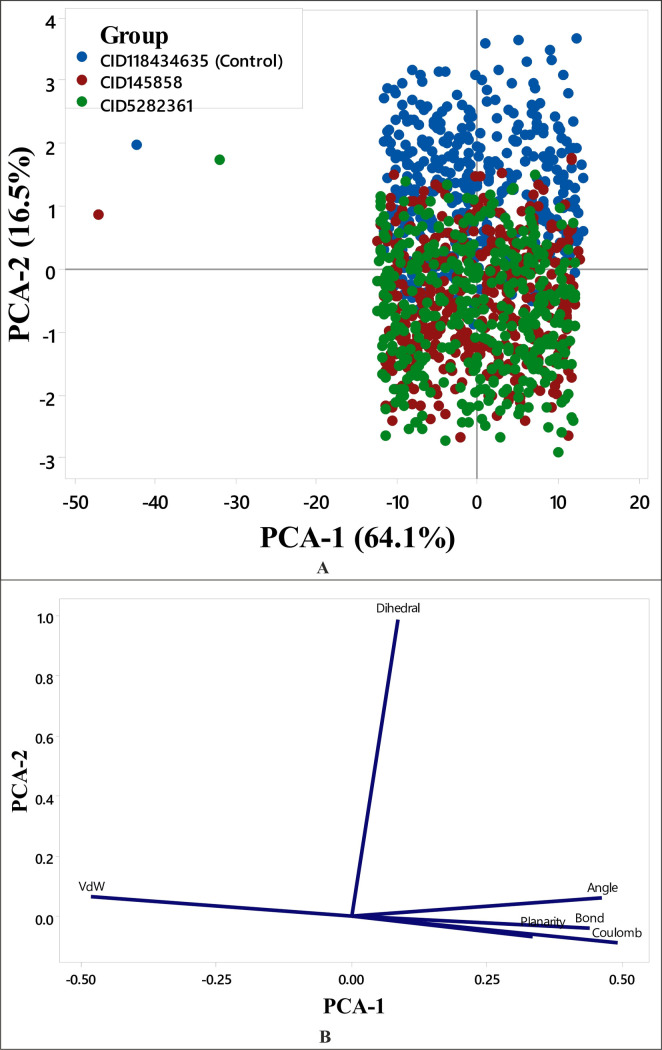
(A) Three data clusters were denoted by different colored dots in the scores plot, where each dot indicates a single time point. (B) Loadings plot of the structural and energy data from Principal Components Analysis.

Principal component analysis often identifies a limited number of principal modes (components) that account for the majority of conformational variability within a dataset. It is widely used to analyze molecular dynamics (MD) trajectories to assess structural similarities and differences among sampled conformations [67]. In this study, PCA was applied to explore long-timescale dynamics of PD-L1 in complex with different ligands. PCA clustering (PC1 and PC2) results were visualized using scatter plots for each replicate. Each circle in the figure (Fig 8A) represents a conformer, with the distribution of circles indicating the conformational changes in the PD-L1 structure upon ligand binding. It was observed from Fig 8A that PCA-1 and PCA-2 collectively account for 80.6% of the variance, where contributions of PCA-1 and PCA-2 were 64.1% and 16.5%, respectively of the fluctuation. The score displayed on the PCA model indicates an overlap between the protein-CID-145858 (red circles) and protein-CID-5282361 (green circles) complexes. Along the positive direction of PCA1-PCA2, the red and green circles form distinct clusters separated from the protein-control cluster, suggesting that ligand binding induces unique and stabilized conformational states. In contrast, the control complex exhibited a more dispersed distribution, indicating higher conformational variability and reduced structural stability. Complementarily, the PCA loading plot (Fig 8B) derived from MD energy profiles and structural data revealed a positive correlation of bond, angle, and Van der Waals (VdW) variables with the ligand-induced conformational shifts, suggesting tighter clusters of PD-L1 complexes bound to CID-145858 and CID-5282361. Furthermore, the clustering in the upper right quadrant of the loading plot represents minor variations in dihedral energy, consistent with the stabilization of the protein-ligand complexes. Additionally, the dPCA (dihedral PCA) results aligned well with the original torsional angle distributions, reinforcing the method’s accuracy in capturing key conformational changes [68].

**Fig 9:**
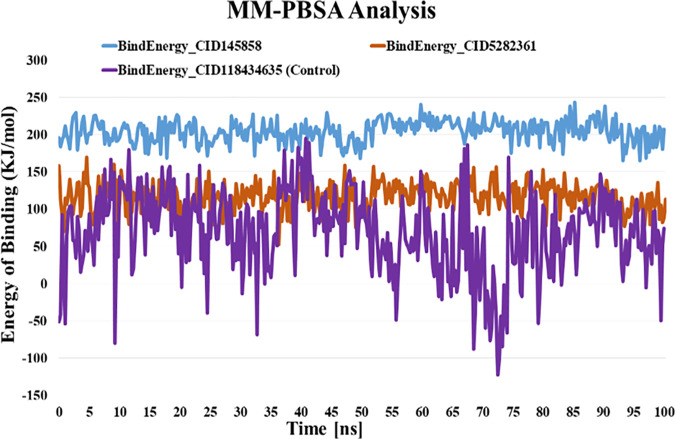
Analyses of binding free energy of Control (BMS-1166) and ligand compounds (CID-145858 and 5282361) complexed with PD-L1 through the MM-PBSA method.

### 3.8. MM-PBSA/energy of binding analysis

The YASARA Stimulator’s MM-PBSA/binding energy analysis and Boundary Quick techniques were used to calculate the binding free energy of the top two ligand-receptor complexes and the control compound-PD-L1 complex. For the MM-PBSA calculations, a brief stable region of 100 ns was extracted from the simulated trajectory of the docked complexes using polar and apolar solvation parameters. PD-L1-CID-145858, PD-L1-CID-5282361, and PD-L1-BMS-1166 complexes were found to have average binding energies of 204.892, 117.003, and 69.364 kJ/mol, respectively. The top two compounds were found to form a stable complex with PD-L1 with substantial binding affinity as determined by the MM-PBSA analysis (Fig 9).

## 4. Discussion

The molecular characteristics and immunogenic nature of TNBC have paved the way for immunotherapy to be considered a promising therapeutic strategy in both advanced and pre-surgical settings. The expression of PD-L1 is observed in approximately 40−65% of TNBC cases and serves as a key predictive biomarker for clinical benefit from anti-PD-L1 monoclonal antibody therapies [69]. However, the comparative efficacy of these small-molecule agents relative to established monoclonal antibodies has yet to be fully characterized, highlighting the necessity for continued rigorous investigation in this area [69–71]. Natural compounds have a longstanding history in the treatment of various human diseases. Notably, approximately 47% of currently available anti-tumor agents are derived from natural sources. Research findings have also indicated that natural compounds have effectively broadened the scope of immunotherapy in treating cold tumors such as TNBC [72]. Therefore, phytochemicals derived from plants are widely considered as viable candidates for cancer treatment [73]. Although the anticancer potential of phytochemicals has been well-documented, their specific effects on the PD-1/PD-L1 interaction remain largely unexplored. In this context, the current study presents a novel investigation into eleven Indian medicinal plants as underexplored sources of PD-L1 inhibitors, highlighting their potential therapeutic role in the treatment of TNBC. While prior research has primarily focused on natural PD-L1 inhibitors, this work uniquely integrates the vast repository of traditional Indian medicinal knowledge with contemporary computational drug discovery approaches, thereby bridging ethnopharmacology and advanced scientific methodologies. Historically, these plants have been predominantly employed worldwide for the management of inflammation and oxidative stress-related disorders [73–80]. Both of these pathological conditions have been associated with the emergence of multiple malignancies, including TNBC. In particular, inflammation has been associated with the increased expression of PD-L1 in TNBC, subsequently restricting the efficacy of ICIs. Furthermore, oxidative stresses may upregulate PD-L1 in tumors via enhanced generation of reactive oxygen species (ROS). Elevated levels of ROS have also been reported in TNBC [81–84].

This study used molecular docking to screen 953 compounds from selected medicinal plants targeting human PD-L1 (PDB ID: 5J89) and BMS-1166 as the reference molecule. Initially, 20 compounds exhibiting higher binding affinities than the control were selected for pharmacokinetic and toxicity analyses. Among these, two compounds - CID-5282361 (Docking score: −9.9 kcal/mol) and CID-145858 (Docking score: −9.5 kcal/mol) demonstrated strong binding affinities toward PD-L1 in molecular docking studies, along with favourable ADMET profiles. Hotspot residues on protein surfaces are crucial for protein-ligand complex formation and serve as key targets in drug design to inhibit protein-protein interactions [85]. In the aim of formulating inhibitors designed to disrupt the PD-1/PD-L1 interaction, several residues of PD-L1, as documented in previous studies, include Tyr 56, Met 115, Ala 121, Asp122-Arg125, Tyr 123, and Ile 54 played significant roles [86–88]. In particular, TYR56 plays a critical role in binding of both antibodies and small molecular inhibitors with PD-L1, whereas MET115 is important for small molecules binding [89]. Investigations have shown that MET115 significantly impacts the conformational state of PD-L1 during its binding to diverse ligands [90]. Analyses of non-bond interactions revealed the presence of both hydrogen and hydrophobic bonds between the protein and the two most promising ligands and the control molecule. Each ligand established hydrophobic bonds with Tyr 56, Met 115, and Ala 121 residues of PD-L1, whereas the control drug interacted with Asp 122, Tyr 123, and Arg 125 residues. Hydrophobic interactions are also reported to amplify the binding affinity between the target and ligand [89]. Structural analysis of human PD-1/PD-L1 demonstrates that both proteins utilize extensive hydrophobic surfaces in their Ig-like V-type domains for ligand interaction. Furthermore, an in-depth analysis of the PD-L1 binding pocket indicates that the tunnel-shaped hydrophobic pocket formed by Tyr56, Met115, Ala121, and Asp122 is significant in ligand binding [86]. PD-L1 mutants like A121Q and Y56A/M115A showed reduced binding to effector cells, highlighting the importance of these residues [91]. Consequently, compounds that establish hydrophobic interactions within this specific region may enhance the inhibitory efficacy of PD-L1 by blocking PD-1/PD-L1 interactions [92]. Hydrophobic interactions are also reported to amplify the binding affinity between the target and ligand [89]. Therefore, our result is in agreement with the previous studies. On the other hand, both compounds exhibited benzene rings, as evidenced by their two-dimensional structural representations. Aromatic rings of compounds were found to enhance the affinity and specificity of drug-like entities by participating in protein-ligand interactions, facilitating non-covalent interactions with hydrophobic residues located within binding sites of the target protein [93,94]. This structural feature is critically important in developing protein kinase inhibitors as aromatic rings correlate positively with binding affinity, balancing the loss of hydrogen bond interactions [94]. In 2023, seven small-molecule protein kinase inhibitors got FDA approval for various cancer types [95]. A study conducted in 2019 further suggested that the occurrence of aromatic rings containing compounds could be anticipated as innovative PD-L1 inhibitors [96].Evaluation of pharmacokinetic and toxicological profiles of the top two lead compounds provided essential insights into their potential efficacy and safety, aiding in the efficient utilization of time and resources [97]. Both selected compounds exhibited lower molecular weight, moderate solubility, and improved gastrointestinal absorption, suggesting their potential suitability as oral drug candidates [89,90]. The toxicity profiles of the lead compounds demonstrate the absence of cytotoxicity and immunotoxicity, both of which were critical factors in the selection of ICI [98,99]. Both these compounds were subjected to 100 ns molecular dynamics simulations to evaluate their stability when bound to their target protein. Although extended simulations may reveal additional insights into conformational changes, we observed stabilization of the system within the 100 ns timeframe of the MD run and successfully fulfilled our objective of identifying the most promising candidates. Furthermore, the 100 ns MD simulation was extensively utilized to evaluate the inhibitory potential of phytochemicals [45,100,101]. Therefore, the MD run was limited to 100 ns. Both complexes exhibited RMSD values converging below 4.0 Å compared to the control complex (approximately 5 Å) after duration of 50 ns. On the other hand, RMSF analysis further demonstrated reduced flexibility (<1.5 Å) for these compounds compared to the control within the hotspot region of PD-L1 (residues 56–66), a finding of particular significance for antibodies and small molecule inhibitors in general [89]. Therefore, RMSD and RMSF from MD simulation further corroborated the stability of both ligand-protein complexes, affirming the inhibitory capabilities against PD-L1. Moreover, both selected compound complexes exhibited a lower mean Rg and reduced flexibility relative to the control complex, suggesting that they promote protein stabilization in a more compact conformation [102]. Consistent with the observed reductions in Rg values, SASA analysis confirmed that the protein expansion upon ligand binding remains within the optimal level. Notably, protein flavylium (CID-145858) complex exhibited the lowest SASA value, indicating minimal solvent exposure. It also demonstrated the highest stability of solute hydrogen bonds and the fewest solvent interactions, suggesting strong and specific binding affinity. This result is consistent with its MM-PBSA binding energy of −204.892 kJ/mol. In contrast, 4-Hydroxychalcone (CID-5282361) showed a slightly higher number of solvent hydrogen bonds, reflecting moderate binding strength with MM-PBSA of −117.003 kJ/mol. The control complex displayed the greatest number of solvent hydrogen bonds, suggesting weak binding specificity, corroborated by its MM-PBSA binding energy of −69.364 kJ/mol. Furthermore, principal component analysis (PCA) revealed more compact conformational clustering for both ligand-bound complexes compared to the control, indicating enhanced structural stability of these two PD-L1 complexes. Based on these findings, the selected two compounds have the potential to serve as lead compounds for inhibiting PD-L1’s biological activity.

## 5. Conclusions

Targeting Programmed death ligand-1 (PD-L1) represents a potential therapeutic strategy to address the challenges associated with TNBC management. Developing a novel small-molecule inhibitor may offer advantages in overcoming the limitations of antibody-based anti-PD-L1 drugs. Therefore, in the present study, we screened 953 new phytochemicals derived from selected Indian medicinal plants to identify anti-PD-L1 compounds for the treatment of TNBC using computational methodologies. The results of molecular docking analysis identified two compounds, 4-Hydroxychalcone (CID-5282361) and flavylium (CID-145858), exhibiting superior binding affinity to the target protein. Subsequent ADME and toxicity evaluations qualified these two compounds as promising drug candidates. Molecular dynamics simulations demonstrated the stable interaction of these 2 compounds with PD-L1 throughout the simulation period. Therefore, our findings suggest that 4-Hydroxychalcon and flavylium derivatives exhibit potential as PD-L1 inhibitors. Nonetheless, further experimental validation is essential to confirm their efficacy in PD-L1 inhibition and to substantiate their suitability as prospective drug candidates for the treatment of TNBC in the future.

Although these *in silico* studies offer a cost-effective and efficient approach to screen potential PD-L1 inhibitors, the precision of outcomes can be constrained by the quality of data and the effectiveness of the computational algorithms employed. Furthermore, the reliability of these predictions can be compromised by the inherent flexibility and flatness of the PD-L1 interacting surfaces [88]. Due to unforeseen biological factors, virtual screening may generate false positives when predicted active compounds fail to demonstrate efficacy in experimental validation [45]. Therefore, to increase the reliability of *in silico* results and support the transition of computational predictions into therapeutic use for TNBC, it is essential to validate these two compounds through wet lab experiments. Techniques such as Western Blot analysis and Flow Cytometry can be employed to assess changes in PD-L1 protein levels and quantify PD-L1 expression on TNBC cells following treatment with two compounds. MTT assay using the MDA-MB-231 cell line can also be used to assess the cytotoxic effects of these identified compounds. Furthermore, using TNBC xenograft models, the anti-tumor efficacy of compounds can be evaluated by monitoring tumor growth and metastasis. Lastly, transcriptomics analyses can be used to assess gene expression changes associated with immune evasion mechanisms.

These findings underscore the importance of integrating computational and experimental approaches in drug discovery. While computational methods offer valuable insights and can accelerate the initial stages of lead compound identification, they must be complemented by rigorous experimental validation to ensure the reliability and efficacy of potential drug candidates. This synergistic approach not only enhances the accuracy of predictions but also addresses the inherent limitations of computational techniques.

It is important to note that despite the advantages of using small molecules for targeted therapy, there are also several challenges. Phytochemicals can engage with various molecular targets, potentially causing unintended side effects that make their development as selective PD-L1 inhibitors more challenging [26]. Even if a phytochemical appears promising, establishing cost-efficient and scalable methods for its extraction or synthesis is crucial for clinical use. On the other hand, patient responses to phytochemical treatments can be affected by genetic and environmental factors, highlighting the need for personalized therapeutic approaches. Most targeted anti-cancer drugs develop resistance after some time. Targeted anticancer drugs also face low efficiency challenges [103]. With a comprehensive grasp of tumor pathology and advancements in new drug research and development technologies, we anticipate that more innovative small-molecule anti-cancer drugs targeting novel genes or mechanisms of action will emerge soon. Additionally, it is predicted that new fields, such as the integration of small-molecule targeted drugs with tumor immunotherapy, antibody-drug conjugate (ADC), and Proteolysis targeting chimeric (PROTAC) technology, will experience significant growth over the next decade.

## Supporting information

S1 Table List of natural compounds and their plant sources showing binding affinity in Kcal/mol with the receptor. (XLSX)

S1 File PDB of top 20 protein-ligand docked complexes along with protein-control docked complex. (ZIP)
